# Oral microbiome dysbiosis in autism spectrum disorder: the oral-gut-brain axis and future perspectives: a narrative review

**DOI:** 10.3389/fmicb.2026.1783810

**Published:** 2026-03-04

**Authors:** YongMao Huang, QiuYing Liang, Youjin Shen, Junjie Chen, Wenan Xu

**Affiliations:** 1 School of Stomatology, Shenzhen Clinical College of Stomatology, Southern Medical University, Guangzhou, Guangdong, China; 2 Department of Pediatric Dentistry, Shenzhen Stomatology Hospital (Pingshan), Southern Medical University, Guangzhou, Guangdong, China; 3 Department of Stomatology, The Third Affiliated Hospital of Southern Medical University, Guangzhou, Guangdong, China

**Keywords:** autism spectrum disorder, metagenomic, microbial biomarkers, multimodal, oral microbiota, oral-gut-brain axis

## Abstract

Autism spectrum disorder (ASD) is a complex neurodevelopmental condition with a steadily increasing global prevalence, yet its etiology remains largely unclear. Emerging evidence suggests that oral microbiome dysbiosis may contribute to the pathogenesis of ASD, potentially through the oral-gut-brain axis, although the exact role and causality remain to be fully established. In this narrative review, we synthesize recent clinical and metagenomic evidence on oral microbiome alterations in ASD and critically evaluate the potential pathways through which these microbial imbalances may impact neurodevelopmental outcomes. We summarize the key host–microbe interactions, including inflammatory signaling, epithelial barrier disruption, and immune-neural crosstalk, while emphasizing that direct causal evidence is still limited. Dysbiosis in individuals with ASD is characterized by altered microbial communities, including increased *Streptococcus* and decreased *Prevotella*, which correlate with clinical symptom severity. Moreover, metagenomic profiling has indicated the presence of potential biomarkers in the oral microbiome, which may serve as promising noninvasive diagnostic tools for ASD. While the clinical applications of oral microbiome diagnostics are still in the early stages, we explore the challenges and opportunities for developing these biomarkers for risk stratification. Finally, we outline future research directions that could enhance the understanding of the oral microbiome’s role in ASD and facilitate the development of personalized intervention strategies.

## 1 Introduction

Autism spectrum disorder (ASD) is a complex neurodevelopmental condition characterized by deficits in social interaction, impaired communication, and repetitive behaviors (American Psychiatric Association, 1980). Despite advances in genetic research, the precise etiology remains poorly understood, underscoring the importance of exploring environmental and microbiome-related mechanisms. The increasing prevalence of ASD worldwide positions it as a significant public health concern, profoundly impacting affected individuals and their families (Lord et al., 2018). Emerging studies highlight microbiota dysbiosis, particularly oral-gut microbial imbalances, as potential contributors to neuroimmune dysregulation in ASD through the oral-gut-brain axis, a bidirectional communication network linking microbial metabolites, systemic inflammation, and neurological function (De Angelis et al., 2013; Needham et al., 2021; Tong et al., 2022; Bundgaard-Nielsen et al., 2023).

This narrative review synthesizes current evidence regarding oral microbial dysbiosis in ASD, emphasizing the “oral-gut-brain axis” as an integrative framework for understanding disease mechanisms. We explore how dysbiosis in oral microbial communities, particularly elevated levels of *Streptococcus* and reduced levels of *Prevotella*, can disrupt gut barrier integrity and facilitate the systemic circulation of neuroactive metabolites, such as lipopolysaccharides (LPS) and short-chain fatty acids (SCFAs). These metabolites may trigger systemic inflammation and subsequently impair neurological function through microglial activation (Gilbert et al., 2016, 2018; Duvallet et al., 2017). While the precise mechanism remains unclear, microbiome imbalances are increasingly recognized as significant contributors to ASD pathology, prompting a shift toward microbiota-based diagnostic and therapeutic strategies. Given the accessibility of the oral microbiome, it offers a unique opportunity for developing noninvasive diagnostic biomarkers. Metagenomic technologies provide precise profiling of microbial dysbiosis and functional metabolic pathways involved in ASD, offering greater insight beyond traditional microbial culture methods. This approach can identify specific microbial biomarkers and elucidate functional pathways potentially associated with ASD pathogenesis. By characterizing these microbial alterations at both the taxonomic and functional levels, metagenomic analysis represents a promising approach to bridge basic research findings with clinical applications.

Furthermore, we examine the emerging role of artificial intelligence (AI), particularly multimodal deep learning models, in integrating metagenomic data with other physiological markers for early ASD diagnosis. Several studies have demonstrated the potential of AI models, such as machine learning classifiers and deep learning algorithms, to predict ASD based on gut microbiome data and physiological imaging (Olaguez-Gonzalez et al., 2023; Nam et al., 2024). In 2025, Temiz et al. used machine learning techniques to predict ASDs on the basis of metagenomic data, identifying microbial features potentially associated with the disorder (Temiz et al., 2025). In another study, machine learning models such as random forests and support vector machines were employed to classify gut microbiome samples from children with ASD and neurotypical controls on the basis of microbial abundance and functional pathways, achieving high classification accuracy (Bašić-Čičak et al., 2024). Moreover, researchers have proposed multimodal deep learning algorithms that integrate functional MRI with gut microbiome data for ASD prediction (Nandhini and Ananthajothi, 2025). These studies exemplify the emerging trend of combining physiological imaging with metagenomic data for AI-based diagnosis, aligning with the future direction of multimodal screening strategies. The application of metagenomics offers high resolution at the species level for microbial identification, providing a promising foundation for developing an accurate and universally applicable multimodal AI model for the early screening of ASDs on the basis of microbial biomarkers. These advancements offer a promising avenue for developing noninvasive, accurate, and widely applicable diagnostic tools.

This review synthesizes current evidence on oral microbial dysbiosis in ASD, with a focus on the “oral-gut-brain axis” as a central framework for understanding disease mechanisms. We also examine how advances in metagenomic technologies and the application of multimodal AI models can help translate microbiome findings into practical clinical tools. We propose that ASD should be viewed not only as a neurodevelopmental disorder but also as a systemic condition, one that may benefit from microbiome-based diagnostic and therapeutic approaches. In addition to the progress in metagenomics, we also explore the potential of multimodal AI models in detecting variations in the oral microbiome to develop early diagnostic and predictive tools for ASD. This approach holds promise for improving the precision and reliability of early ASD detection, ultimately enabling more personalized and effective interventions. This emerging paradigm suggests that a deeper, system-wide understanding of ASD, informed by the oral-gut-brain axis and microbiome changes, may lead to more comprehensive and impactful clinical strategies for early detection and management.

## 2 The social burden of autism and the potential of oral microbiota diagnostics

The prevalence of ASD has markedly increased over recent decades (Baribeau et al., 2022; Lage et al., 2024). Despite advancements in genetic diagnostics, definitive etiological explanations remain elusive, underscoring the necessity of exploring environmental and microbiome-driven mechanisms. The global prevalence of ASD has markedly increased, with the 2023 Centers for Disease Control and Prevention (CDC) Community Report on Autism indicating an increase in diagnosis rates from 1 in 88 children (1.1%) in 2008 to 1 in 36 children (2.76%) in 2020, representing a 151% increase over 12 years (Hirota and King, 2023; Maenner, 2023). ASD affects approximately 1 in 100 children worldwide (Plaza-Diaz et al., 2022), although regional disparities persist, such as lower rates in Shanghai, China (8.3 per 10,000) (Jin et al., 2018), than in 59 children in the United States (Olsen and Hicks, 2020). Owing to the behavioral challenges inherent in autism, many patients initially experience difficulties in cooperating with outpatient treatment. In light of the rising prevalence of autism, developing an efficient, accurate, and cost-effective early screening and diagnostic tool has become an urgent global challenge that needs to be addressed.

Currently, diagnosis relies predominantly on behavioral indicators and standardized assessments such as the Autism Diagnostic Interview (ADI) and Autism Diagnostic Observation Schedule (ADOS) (Sanders, 2019). Despite their effectiveness, these tools carry a risk of misdiagnosis owing to their subjective nature, emphasizing the importance of developing more objective diagnostic biomarkers. Advanced diagnostic approaches frequently incorporate neuropsychological assessments combined with neuroimaging techniques, such as electroencephalography (EEG) and magnetoencephalography (MEG). The Diagnostic and Statistical Manual of Mental Disorders, Fifth Edition (DSM-5), categorizes ASD severity into three distinct levels: mild, moderate, and severe.

The limitations of current diagnostic methods highlight the potential for microbiome-based diagnostics, specifically through profiling oral microbiota dysbiosis. This approach may provide noninvasive and objective diagnostic biomarkers, paving the way for novel clinical interventions. There is emerging evidence suggesting that oral microbiota dysbiosis, including an overabundance of *Streptococcus* species, could be involved in linking oral health issues with ASD, though the exact mechanisms remain under investigation (D’Angelo et al., 2025). Compared with the general population, individuals with ASD exhibit a significantly greater prevalence of oral diseases (Ferrazzano et al., 2020). The differences in oral health between individuals with autism and healthy populations suggest that focusing on the oral health of individuals with autism, such as monitoring the dynamic changes in oral microbiota, may offer a new perspective for identifying potential early screening and diagnostic biomarkers for autism.

## 3 Gut microbiome changes in patients with autism spectrum disorders

Many research methodologies have been employed in an attempt to explore the pathogenesis of autism. However, the precise etiological mechanisms remain ambiguous due to the intricacy of the underlying factors. Initially, the hypothesis that environmental factors play a primary role in the etiology of ASD was once predominant (Modabbernia et al., 2017). Nevertheless, recent research has demonstrated that the etiology of ASD is a multifaceted interaction of environmental, genetic, and neurodevelopmental factors (Garcia-Gutierrez et al., 2020). In addition to the aforementioned factors, other potential etiological factors, such as nutritional deficiencies, viral exposure, and immune system dysfunctions, have also been identified (Risch et al., 2014; Doernberg and Hollander, 2016). Genetic studies have identified hundreds of genes linked to an increased risk of ASD, thereby deepening our understanding of genetics in its pathogenesis (Kraneveld et al., 2016; Li et al., 2017). Together, these studies highlight the complexity of ASD pathogenesis, indicating that its development is shaped by a mix of genetic, environmental, with growing interest in microbiological factors as potentially modifiable contributors.

Recent studies have increasingly recognized gut microbiota dysbiosis as a susceptibility factor for neurological disorders, including ASD, Alzheimer’s disease, Parkinson’s disease, multiple sclerosis, and stroke (Cryan et al., 2020). This link is supported by evidence showing that gastrointestinal symptoms and intestinal dysbiosis are prevalent among individuals with ASD (Fattorusso et al., 2019). Studies of ASD-associated gastrointestinal conditions have reported significant alterations in the gut microbial composition. For example, in children with ASD and gastrointestinal symptoms, a substantial increase in *Bacteroidetes*, particularly *Bacteroides vulgatus*, was observed compared with that in controls, whereas *Firmicutes* dominated in the control groups. Additionally, *Desulfovibrio* species are significantly enriched in ASD stool samples (Finegold et al., 2010). Kang et al. reported a reduction in *Prevotella*, *Coprococcus*, and *unclassified Veillonellaceae* in the microbiota of individuals with ASD (Kang et al., 2013). These findings collectively underscore the relationship between gut microbiota dysbiosis and ASD pathology. Further evidence linking microbiota dysbiosis and ASD pathogenesis involves alterations in microbial pathways related to neurotransmitter synthesis. Significant microbial composition shifts and depleted neurotransmitter biosynthesis pathways have been documented, and specify bacterial species have been regarded as potential diagnostic biomarkers for ASD (Xu et al., 2022; Meng et al., 2024). These microbial imbalances appear to influence the gut-brain axis, a critical communication network connecting gastrointestinal health and neurological functions (Mayer et al., 2015; Cryan et al., 2019). The “gut-brain axis” represents a bidirectional communication network linking the gastrointestinal tract and the central nervous system. This axis significantly influences neurodevelopment via neuroendocrine, immune-mediated, and neural pathways. Microbial metabolites, such as SCFAs, immune interactions, and neural signals, are key components of this complex network (Wang and Kasper, 2014; Dinan and Cryan, 2015). Dysbiosis, characterized by microbiota imbalance, has been associated with disrupted myelination, impaired neural function, and altered neurodevelopment, highlighting the potential relevance of the microbiota to brain health. Consistent with this view, microbiota imbalance has also been linked to both neurodevelopmental and neurodegenerative conditions, supporting interest in the microbiota as a potential therapeutic target (Sharon et al., 2016; Cryan et al., 2019).

Therapeutic approaches targeting gut dysbiosis, such as oral lyophilized fecal microbiota transplantation (FMT), have shown promise in improving gastrointestinal and behavioral symptoms associated with ASD (Li et al., 2024). Preclinical animal studies further support a potential contributory role for gut microbiota dysbiosis in ASD-like behaviors. For example, a study published in *Cell* demonstrated that core ASD behavioral phenotypes, including social impairments and repetitive behaviors, could be induced in germ-free (GF) mice through fecal microbiota transplantation from ASD donors. Supporting mechanistic plausibility, researchers have also reported behavioral symptoms, altered gut barrier integrity, and microbiota disruptions in maternal immune activation mouse models of ASD. Specifically, GF mice exhibit persistent blood–brain-barrier (BBB) permeability, which is reversible upon microbial colonization, and supplementation with *Bacteroides fragilis* significantly alleviates ASD symptoms (Hsiao et al., 2013).

Collectively, these studies have reported differences in the gut microbiota and metabolite profiles between individuals with ASD and neurotypical individuals (De Angelis et al., 2015; Kong et al., 2019). While findings across cohorts remain heterogeneous, the available evidence is broadly consistent with the gut-brain-microbiota axis hypothesis and suggests that microbiota dysbiosis may be relevant to ASD-associated phenotypes. These insights motivate continued investigation of microbiota-focused diagnostics and interventions and provide a rationale for extending the discussion to the oral-gut-brain microbial axis in ASD.

## 4 Oral microbiome changes in patients with autism spectrum disorders

The oral cavity is one of the largest microbial reservoir in the body and hosts diverse microbial communities, including bacteria, fungi, viruses, and archaea, with over 700 identified microbial species (Proctor et al., 2018; de Jesus et al., 2020; Kahharova et al., 2020). These communities maintain a delicate equilibrium between symbiosis and antagonism through mechanisms such as symbiotic adaptation and competitive exclusion (Jenkinson, 2011; Kaan et al., 2021). Disruption of this microbial balance may lead to both oral and systemic diseases, emphasizing the importance of in-depth research into oral microbial ecology and its health implications. In the late nineteenth century, Willoughby D. Miller proposed the concept of oral focal infection (Miller, 1891). The oral cavity, which serves as the primary entry point for the respiratory and digestive systems, acts as a crucial interface connecting oral and systemic health. Consequently, understanding oral microbial interactions is vital for exploring broader implications in systemic diseases, including neurological disorders. Consistent with this view, recent syntheses further highlight that ASD-related behaviors such as sensory sensitivity, food selectivity, and self-injurious behaviors can interact with oral health and, potentially, oral microbial ecology, supporting a bidirectional framework that warrants mechanistic testing (D’Angelo et al., 2025).

**Figure 1:**
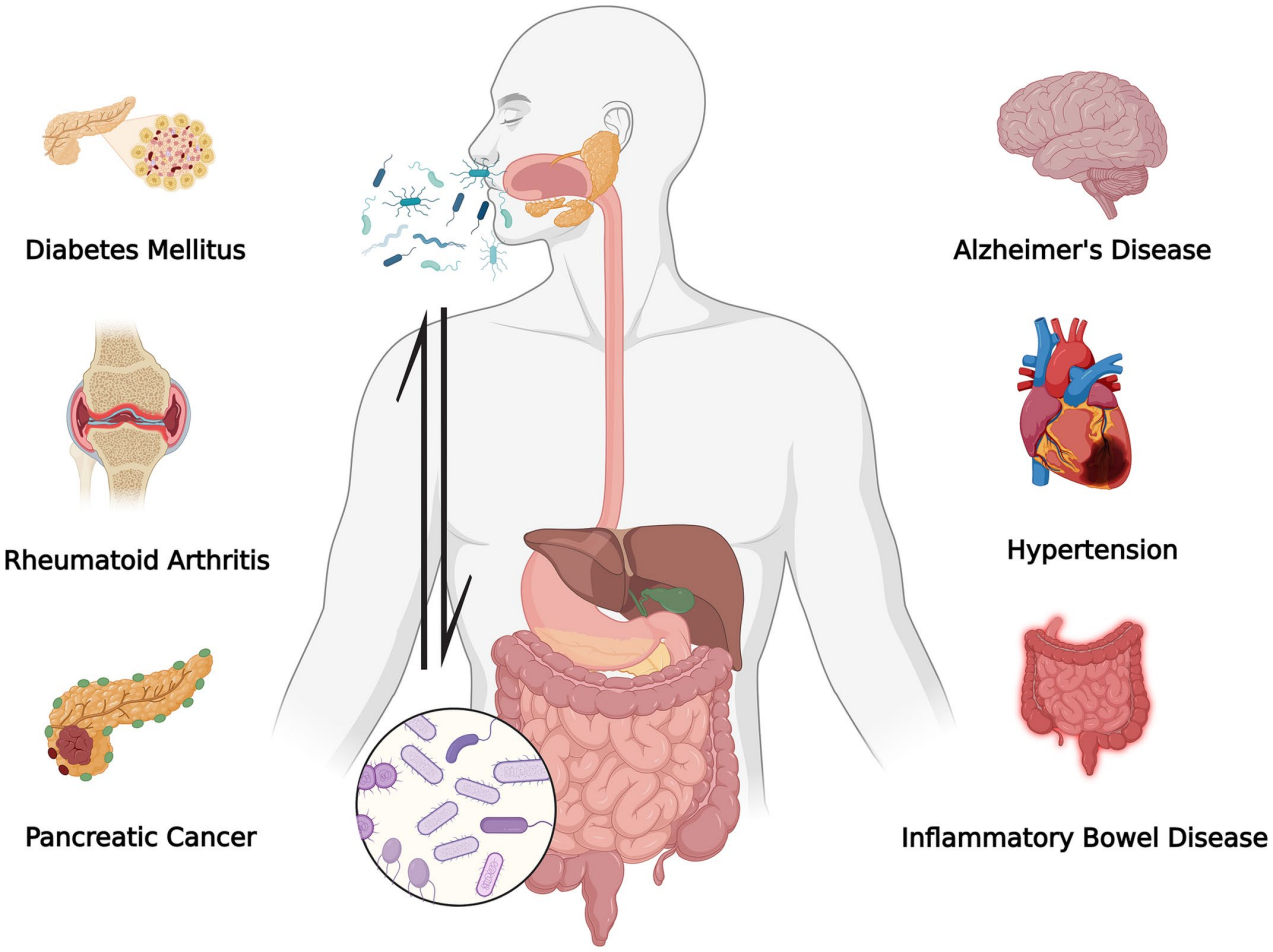
Microbiota dysbiosis and systemic diseases. The potential systemic impact of the oral microbiota on various diseases. Oral bacteria can migrate to the gut, affecting the gut microbiome and influencing the development of several diseases, including diabetes mellitus, rheumatoid arthritis, Alzheimer’s disease, hypertension, and pancreatic cancer. Microbe migration may have far-reaching effects on systemic health. Created with BioRender.com.

Recent research has underscored the potential of imbalances in oral bacteria as a contributing factor to neurological disorders, with studies suggesting that oral microbiota alterations may influence neuroinflammatory and neuropsychiatric processes (Borrego-Ruiz and Borrego, 2025). For example, chronic oral diseases, notably periodontitis, are associated with neurodegenerative disorders. Periodontal pathogens are implicated in Alzheimer’s disease through mechanisms involving systemic inflammation and disruption of neural pathways (Olsen and Singhrao, 2015; Ranjan et al., 2018). Moreover, disturbances in the oral microbiota may impair gut barrier integrity by reducing beneficial microbial metabolites, such as butyrate, compromising communication within the gut-brain axis, and potentially exacerbating neurological conditions, including ASD (Chen et al., 2023). Consequently, therapeutic strategies targeting oral microbial homeostasis could represent a potential adjunctive direction for neurological disorders. Furthermore, imbalances within the oral microbiome have been linked with various systemic diseases beyond neurological conditions (Figure 1) (Tong et al., 2019; Read et al., 2021; Tuominen and Rautava, 2021; Kunath et al., 2022; Peng et al., 2022; Chen et al., 2023; Guo et al., 2023). Certain oral microorganisms can modulate inflammatory responses and serve as diagnostic biomarkers for systemic diseases (Graves et al., 2019). Emerging evidence suggests that oral microbiota alterations are associated with ASD and may relate to ASD-relevant host pathways such as immune and metabolic signaling (Fakhruddin et al., 2025). Against this background, a growing number of studies have examined whether oral microbiota alterations are associated with ASD and related phenotypes. In 2022, researchers reported increased streptococcal phages and virulence factors in the supragingival plaque of ASD individuals compared with age-matched neurotypical controls. These bacterial changes, particularly elevated *Streptococcus* abundance and decreased *Prevotella* clusters, are positively correlated with ASD severity, suggesting a unique oral-brain pathogenic link distinct from that in gut ecosystems (Tong et al., 2022). Similarly, another study demonstrated concurrent alterations in the salivary microbiome composition and microRNA profiles. These microbiota-microRNA shifts are significantly correlated with ASD-related social and communicative impairments, suggesting that saliva may represent a potential noninvasive biomarker (Ragusa et al., 2020). Furthermore, a study reported marked oral microbiota dysbiosis in ASD children, characterized by elevated *Streptococcus* spp. abundance and disrupted *Prevotella*-driven symbiotic networks (Qiao et al., 2018). These microbial alterations are strongly correlated with clinical severity and the incidence of dental caries, paralleling previously reported findings of gut microbiota dysbiosis in individuals with ASD (Kang et al., 2013; Finegold et al., 2017). To explore mechanistic plausibility, Qiao et al. used an oral microbiota transplantation approach by transplanting the oral microbiota of ASD patients into GF mice. Recipient mice exhibited hallmark ASD behaviors, accompanied by notable changes in both oral and intestinal microbiota composition, including shifts in *Bacteroides* and *Porphyromonas* abundance. Concurrently, altered serotonin signaling and activation of the TGF-β pathway, which is strongly implicated in ASD pathophysiology, have been documented in the prefrontal cortex (Qiao et al., 2022). Besides that, recent pediatric case control studies have reported mixed patterns at the community level study, observed no significant group differences in α/β diversity or phylum/genus-level abundances, yet several OTUs were enriched in ASD, underscoring potential subtle, context-dependent shifts. Taken together, these human and animal studies support the plausibility that oral microbial communities can influence host neuroimmune/neurochemical pathways; however, translating these observations into consistent, clinically actionable mechanisms requires further longitudinal and mechanistic validation.

In addition, recent large-scale metagenomic analysis of saliva from more than 2,154 children with ASD revealed distinct oral microbiome profiles that differentiated ASD from neurotypical siblings and correlated with clinical phenotypes (Manghi et al., 2024). Complementing saliva-based work, a dental plaque study in preschoolers reported lower bacterial diversity in ASD and identified a set of discriminatory species; a random-forest model achieved an AUC of 0.937 (95% CI: 0.82–1.00) in leave-one-out cross-validation, emphasizing the promise, but also the need for external validation and prospective testing—of plaque-derived microbial signatures for early-risk stratification (Tang et al., 2025). Further exploratory research revealed that certain genera were enriched in the oral microbiomes of ASD children, with these shifts strongly associated with social impairments, repetitive behaviors, and anxiety symptoms (Evenepoel et al., 2024). Several mechanisms have been proposed to link oral dysbiosis with ASD-relevant phenotypes. One potential mechanism involves altered immune responses mediated by the oral microbiome. Dysbiosis in the oral cavity may lead to increased release of proinflammatory cytokines, such as TNF-α and IL-6, which can disrupt neuroimmune interactions and impair blood–brain barrier function. Oral microbial imbalance may thus trigger systemic inflammatory responses that influence neural development and behavior, which is consistent with evidence linking heightened proinflammatory cytokine profiles to neurodevelopmental disruptions (Cao et al., 2021). These inflammatory cytokines, which are produced locally in the oral cavity and the gut, can activate immune cells such as microglia in the brain, leading to an inflammatory cascade that alters brain structure and function (O’Riordan et al., 2025). This process can result in neurological impairments, including changes in synaptic function, neurogenesis, and neuroplasticity, all of which are associated with ASD symptoms. Experimental models have provided further support, showing that the disruption of specific oral bacteria can induce anxiety-like behaviors in mice, suggesting a biological basis for microbe-mediated behavioral effects (Li B. et al., 2025). Clarifying these underlying microbial and metabolic mechanisms will further elucidate the potential therapeutic role of the oral microbiota in managing ASD symptoms (Mussap et al., 2023).

Moreover, oral microbiota dysbiosis is increasingly recognized as a potential contributor to oxidative stress in individuals with ASD. Disruption of the oral microbial community can lead to an overproduction of reactive oxygen species (ROS) and other oxidative markers, which, when compounded with systemic inflammation, may impair neurodevelopmental processes (de Oliveira et al., 2025). Recent studies have indicated that microbial imbalance in the oral cavity may directly affect redox homeostasis, triggering oxidative damage that influences brain function (Zhong L. et al., 2025). These oxidative processes are linked to neuroinflammation, which plays a critical role in the pathophysiology of ASD. Systemic redox imbalance has been repeatedly reported in ASD across peripheral biospecimens, supporting oxidative stress as a convergent biochemical feature in at least a subset of individuals (Chen et al., 2021). At the cellular level, immune cells from ASD cohorts show heightened TLR4-NOX2 signaling and increased reactive oxygen species generation, and these responses can be further amplified by lipopolysaccharide stimulation, providing mechanistic support for microbe-linked oxidative inflammation (Nadeem et al., 2017). In the oral compartment, emerging case–control evidence suggests that ASD-associated oral microbiota shifts may coincide with altered antioxidant readouts in saliva and oxidative DNA damage signals in oral epithelial cells, although causality remains to be established (Pangrazzi et al., 2025). Oxidative stress and LPS-driven inflammation are also known to compromise blood–brain barrier integrity and promote neuroinflammatory cascades, offering biological plausibility for downstream neurodevelopmental effects (Peng et al., 2021). These findings suggest that targeting oxidative stress through modulation of the oral microbiome could offer novel therapeutic strategies for managing ASD symptoms. Further work may help clarify whether redox-related readouts can serve as informative biomarkers or intervention targets in specific ASD subgroups.

In addition to the previously discussed mechanisms, glial cell involvement is another critical pathway through which oral microbiota dysbiosis may contribute to ASD pathogenesis. Glial cells, including microglia, astrocytes, and oligodendrocytes, play essential roles in maintaining brain homeostasis, regulating inflammation, and supporting neuronal function. Microglia actively engulf synaptic material during postnatal development, and complement-linked signaling has been shown to regulate activity-dependent pruning *in vivo* (Paolicelli et al., 2011). Microbiome perturbations may lead to the release of inflammatory mediators that activate these glial cells, triggering an inflammatory cascade within the brain (Kim, 2024). Microglial activation, in particular, has been associated with neurodevelopmental disorders, including ASD, and is known to affect synaptic pruning, neurogenesis, and neuronal plasticity. Owing to the close connection between the oral and gut microbiota, oral microbial dysbiosis may indirectly influence neuroglial interactions through the oral-gut-brain axis, resulting in abnormal brain development and altered neural function, which could contribute to the core symptoms of ASD, such as social impairments and repetitive behaviors (Camberos-Barraza et al., 2024). Moreover, germ-free and antibiotic-perturbed models provide causal evidence that microbial communities shape microglial maturation and innate immune function, and microbiota depletion has been linked to increased BBB permeability (Erny et al., 2015). While direct oral-specific mechanistic evidence in ASD remains limited, experimental studies show that periodontal bacteria can directly activate microglia and induce neuroinflammatory transcriptional programs, providing biological plausibility that oral dysbiosis and its inflammatory signals could converge on glial pathways relevant to neurodevelopment (Magnusson et al., 2024). Understanding how the oral microbiota influences glial cell activation and subsequent neuroinflammation is crucial for identifying new therapeutic strategies aimed at modulating glial activity and improving ASD outcomes.

In summary, available evidence suggests that oral microbiota alterations are associated with ASD and may intersect with immune, metabolic, and neurobiological pathways relevant to the proposed oral-gut-brain axis. However, systematic synthesis indicates that reported shifts are often subtle and sometimes inconsistent across studies, and that direct links between oral microbial features and core ASD symptoms remain insufficiently substantiated, highlighting the need for longitudinal designs, standardized protocols, and integrated multi-omics with deep behavioral phenotyping. In recent years, several studies have highlighted the potential of the oral microbiome as a source of candidate biomarkers for ASD. These findings are summarized in the Table 1. Collectively, these studies motivate further evaluation of whether salivary or plaque microbial signatures alone or in combination with clinical and multi-omics features can support risk stratification or adjunctive screening in well-designed, externally validated cohorts (Tuganbaev et al., 2022).

**Table 1:** Summary of reported oral microbial alterations in autism spectrum disorders.

Microbial taxon	Sample type	Direction in ASD vs. controls	Key note	References
*Haemophilus*	Saliva	Increased	Significantly higher	Qiao et al. (2018)
*Prevotella*	Saliva and plaque	Decreased	Co-occurring taxa decreased	
*Selenomonas*				
*Actinomyces*				
*Porphyromonas*				
*Fusobacterium*				
*Streptococcus*	Plaque	Increased	Significantly higher	
*Rothia*	Saliva	Increased	Abundance increased	Ragusa et al. (2020)
*Filifactor*				
*Actinobacillus*				
Weeksellaceae				
*Ralstonia*				
Pasteurellaceae				
*Aggregatibacter*				
*Tannerella*		Decreased	Abundance decreased	
*Moryella*				
*Saccharibacteria* TM7-3				
*Microbacterium flavescens*	Plaque	Increased	Detected in ASD plaque	Tang et al. (2025)
*Leptotrichia* sp. HMT-212				
*Prevotella jejuni*				
*Capnocytophaga leadbetteri*				
*Leptotrichia* sp. HMT-392				
*Porphyromonas* sp. (HMT-278)				
*Fusobacterium nucleatum* subsp. *polymorphum*		Decreased	Detected in TD plaque; lower in ASD	
*Schaalia* sp. HMT-180				
*Leptotrichia* sp. HMT-498				
*Actinomyces gerencseriae*				
*Campylobacter concisus*				
*Actinomyces odontolyticus*	Tongue	Increased	Enriched	Abdulhaq et al. (2021)
*Actinomyces lignnae*				
*Campylobacter concisus*		Decreased	Depleted	
*Streptococcus vestibularis*				
*Solobacterium*		Increased	Significantly more abundant	Evenepoel et al. (2024)
*Stomatobaculum*				
Ruminococcaceae UCG-014				
*Tannerella*				
*Campylobacter*				

ASD, Autism Spectrum Disorder; TD, Typically Developing; HMT, Human Microbial Taxon.

## 5 Oral-gut microbial transfer and shared signals

The oral cavity itself functions as a dynamic ecosystem. Importantly, the oral and intestinal microbiomes are not independent niches, studies have shown that oral microorganisms may translocate to the large intestine, where they can be detected and, under certain conditions, may colonize the gut, even in healthy individuals (Gao et al., 2018; Azzolino et al., 2025). Accumulating evidence suggests that commensal oral bacteria can reach the gastrointestinal tract via both hematogenous dissemination and enteral passage. Such oral-to-gut microbial dissemination has been implicated in aggravating multiple gastrointestinal disorders, including irritable bowel syndrome, inflammatory bowel disease, and colorectal cancer (Kitamoto et al., 2020). This highlights the complex relationship between the oral and intestinal microbiomes. Such translocation has been proposed as one route by which oral dysbiosis could influence systemic inflammation and downstream neuroimmune signaling, thereby motivating investigation of oral microbial contributions to neurological conditions (Yamazaki and Kamada, 2024). The oral cavity and the gastrointestinal tract are interconnected through the digestive system, facilitating microbial transfer via two primary pathways: the enteric route, where oral microbes transit through the gastrointestinal tract, and the hematogenous route, in which bacteria enter the bloodstream and potentially colonize gut sites (Kunath et al., 2024). Although the role of the oral microbiota in the pathogenesis of ASD is still an emerging area of research, recent studies suggest several key mechanisms by which oral dysbiosis could contribute to neurodevelopmental and neuroimmune disruptions. One of the primary mechanisms proposed is the oral-gut-brain axis, where dysbiosis in the oral microbiome could influence the gut microbiota composition, thereby affecting gut integrity, permeability, and systemic immune responses (Fakhruddin et al., 2025). In line with this framework, oral dysbiosis has been proposed as a potential upstream driver of gastrointestinal dysbiosis and inflammatory signaling with downstream effects on the brain. Under conditions that compromise protective barriers such as gastric acid, bile, or resident intestinal microbiota, oral bacteria can establish ectopic colonies in the gut (Valles-Colomer et al., 2023).

Moreover, oral bacterial migration to the gut may increase intestinal permeability, facilitating the systemic entry of neuroactive metabolites, including LPS, SCFAs, and inflammatory cytokines. SCFAs have been shown to impact neurotransmitter synthesis and release, influencing neural activity and behavior, and may interact with inflammatory cytokine signaling (O’Riordan et al., 2022). For example, butyrate, a key SCFA, has been implicated in regulating neuroinflammation and promoting the production of brain-derived neurotrophic factor (BDNF), a critical factor for neurodevelopment and synaptic plasticity. Consistent with the relevance of SCFA perturbations in ASD, Liu et al. reported lower fecal acetate and butyrate levels and higher valeric acid in Chinese children with ASD compared with neurotypical controls, alongside reductions in multiple butyrate-associated taxa (Liu et al., 2019). Notably, oral-gut crosstalk has also been discussed in relation to SCFA perturbations and broader epigenetic-relevant pathways within the oral-gut-brain axis (Mostafavi et al., 2025). These alterations may compromise BBB integrity, disrupt normal neurodevelopmental processes in the prefrontal cortex, and thereby contribute to core ASD symptoms such as social impairment and repetitive behaviors (Qiao et al., 2022). These findings are supported by previous research, which highlights the role of the oral microbiota in neurodevelopmental disorders (Macfabe, 2012; de Theije et al., 2014).

**Figure 2:**
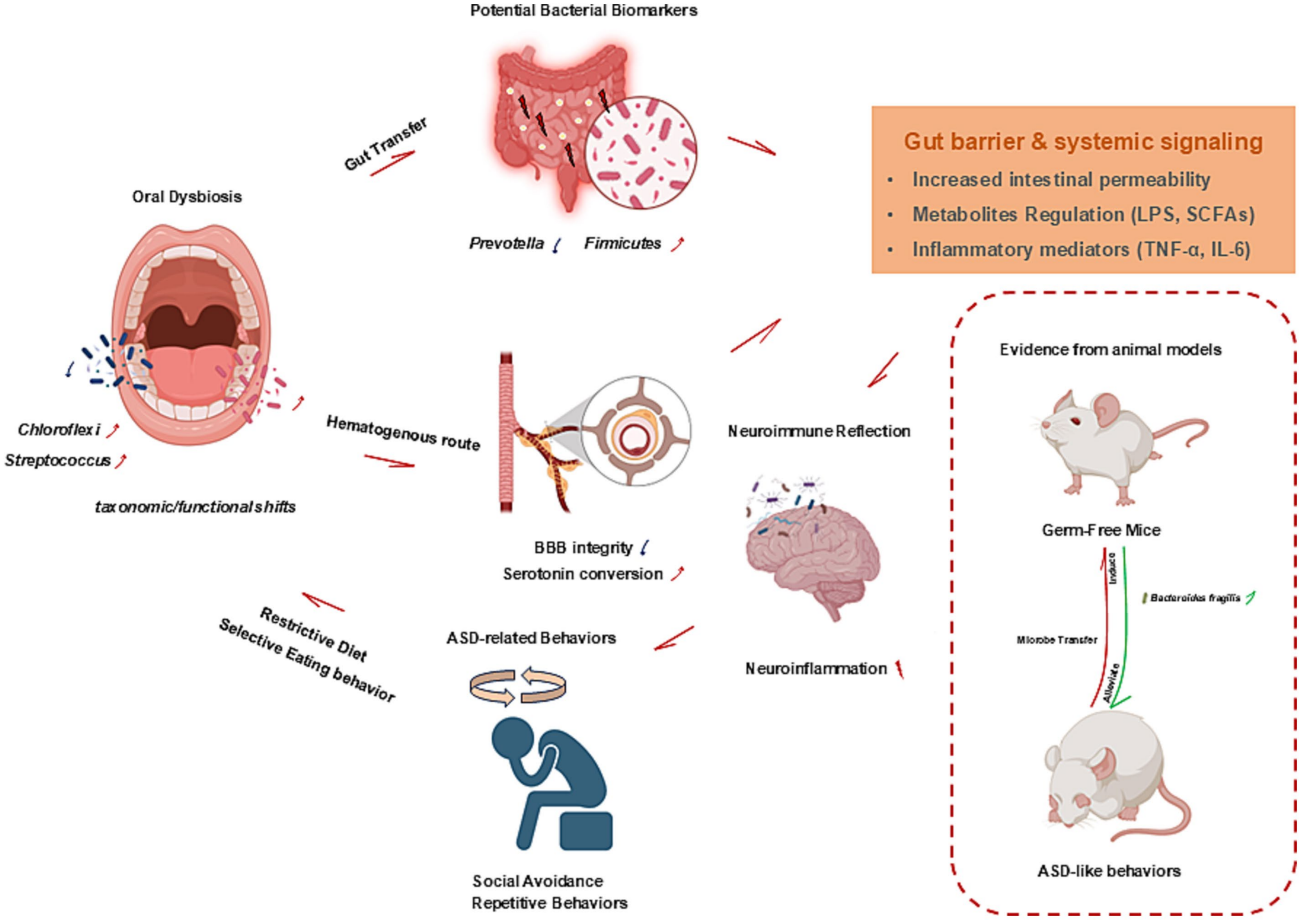
Potential mechanisms linking the oral microbiota to ASD. Oral microbes may reach the gut via enteral transit or hematogenous dissemination and, under permissive conditions, contribute to gut microbial imbalance. Downstream host responses may involve altered intestinal barrier function, systemic inflammatory signaling, and shifts in microbial metabolites. These peripheral changes are proposed to influence neurodevelopment through neuroimmune and neurochemical processes, including potential effects on BBB function and microglial activation. An inset summarizes evidence from animal models supporting a causal contribution of microbiota transfer to ASD-like behaviors. ASD, autism spectrum disorder; BBB, blood–brain barrier; GF, germ-free; SCFAs, short-chain fatty acids; LPS, lipopolysaccharide; TNF-α, tumor necrosis factor-α; IL-6, interleukin-6; TGF-β, transforming growth factor-β.

To date, only a limited number of studies have profiled oral and fecal microbiomes in parallel within the same ASD cohorts. For instance, paired profiling of saliva and stool has been used to explore candidate oral and gut microbial features relevant to ASD stratification and biomarker discovery. Recent oral- and gut-derived microbiome studies in ASD, including sample types, cohort sizes, analytical approaches, and key findings, are summarized in Table 2. In a paired saliva-stool ASD cohort, Kong et al. reported a cross-site signal in which higher gut *Firmicutes* tracked with higher salivary *Chloroflexi* in ASD but not in controls, suggesting a potential oral-gut overlap useful for biomarker discovery (Kong et al., 2019). For example, metagenomic analysis of supragingival plaque revealed enrichment of streptococcal phages and virulence factors and reported correlations with ASD severity, however, analogous severity-linked signals were not observed in fecal samples, underscoring potential site specificity, while still suggesting partial oral-to-gut microbial transmission at the phage level (Tong et al., 2022). At the taxonomic level, reduced *Prevotella* related features have been reported in the gut of autistic children and have also been observed as reduced *Prevotella* clusters in oral datasets (Kang et al., 2013). Accumulating evidence implicates microbial influences on nervous system development and immune regulation through bidirectional host–microbe interactions (Ahrens et al., 2024). Research on the gut microbiota has shown that microbial communities, including fungal and viral components, can modulate immune pathways and neurobehavioral phenotypes, highlighting the broader relevance of host-microbe crosstalk in neurodevelopment. This concept is supported by evidence that gastrointestinal symptoms and intestinal dysbiosis are prevalent among individuals with ASD, with consistent alterations in gut microbial composition observed in clinical studies (Fattorusso et al., 2019). For example, in children with ASD and gastrointestinal symptoms, a substantial increase in *Bacteroidetes*, particularly *Bacteroides vulgatus*, was observed compared with that in controls, whereas *Firmicutes* dominated in the control groups. Additionally, *Desulfovibrio* species are significantly enriched in ASD stool samples (Finegold et al., 2010). We therefore propose a working “oral-gut-brain” framework in which oral microbes may reach the gut through enteral or hematogenous routes and, under permissive conditions, contribute to gut dysbiosis. Downstream changes in gut barrier function and systemic signaling could then influence neuroimmune processes relevant to ASD (Figure 2).

**Table 2:** Systematic overview of oral- and gut-derived microbiome evidence in autism spectrum disorders.

Subject	Title	Sample type	Study characteristics		Key finding	Specify microbial changes	
Finegold et al. (2010)	Pyrosequencing study of fecal microflora of autistic and control	Fecal	Pyrosequencing Sample size: *N* = 48	33 with ASD with GS; 7 sibling controls; 8 non-sibling controls	The abundance of *Bacteroidetes* and *Firmicutes* at the phylum level showed the greatest differences between autism groups of varying severity *Desulfovibrio* spp. and *Bacteroides vulgatus* were present in significantly higher numbers in stools of severely autistic than in controls	*Bacteroidetes ↑* *Firmicutes ↑* *Desulfovibrio* spp. *↑* *Bacteroides vulgatus ↑*	
Kang et al. (2013)	Reduced incidence of *Prevotella* and other fermenters in intestinal microflora of autistic	Fecal	16 s rRNA Sample size: *N* = 40	20 neurotypical and 20 ASD	Significantly lower abundances of the genera *Prevotella*, *Coprococcus*, and unclassified *Veillonellaceae* in autistic samples Changes in the overall diversity and individual species richness of autism-associated gut microbial communities are associated with the presence of autism symptoms, but not with their dietary patterns	*Prevotella ↓* *Coprococcus ↓* *Unclassified Veillonellaceae ↓*	
Kang et al. (2017)	Microbiota Transfer Therapy alters gut ecosystem and improves gastrointestinal and autism symptoms: an open-label study	Fecal	16 s rRNA Sample size: *N* = 18	18 children with ASD	FMT treatment may alter gut microbiota and enteroviruses to improve gastrointestinal and behavioral symptoms of ASD. And maintains this improvement after treatment ends MTT treatment can reverse gut dysregulation mediated by phages in individuals with ASD	*/*	
Qiao et al. (2018)	Alterations of oral microbiota distinguish children with autism spectrum disorders from healthy controls	Saliva; plaque	16 s rRNA Sample size: *N* = 59	32 children with ASD and 27 healthy controls	Pathogens such as *Haemophilus* in saliva and *Streptococcus* in plaques showed significantly higher abundance in ASD patients Commensals such as *Prevotella*, *Selenomonas*, *Actinomyces*, *Porphyromonas*, and *Fusobacterium* were reduced	*Haemophilus ↑* *Streptococcus ↑* *Pathogens:* *Haemophilus ↑*	*Prevotella ↓* *Selenomonas↓* *Actinomyces↓* *Porphyromonas↓* *Fusobacterium↓* *Prevotella↓* *Selenomonas↓* *Actinomyces↓* *Porphyromonas↓* *Fusobacterium↓*
Hicks et al. (2018)	Oral microbiome activity in children with autism spectrum disorder	Saliva	16 s rRNA Sample size: *N* = 346	ASD, *n* = 180; TD, *n* = 106; DD, *n* = 60	12 taxa were altered between the developmental groups and 28 taxa were identified that distinguished ASD patients with and without GI disturbance Five microbial ratios distinguished ASD from TD participants (79.5% accuracy), three distinguished ASD from DD (76.5%), and three distinguished ASD children with/without GI disturbance (85.7%) Significant differences within energy metabolism and lysine degradation	*/*	
Ma et al. (2019)	Altered Gut Microbiota in Chinese with Autism Spectrum Disorders	Fecal	16 s rRNA Sample size: *N* = 90	45 ASD and 45 TD	The abundance of microbiota at the phylum level showed no differences between the autism groups and the healthy control At the family level, ASD exhibited lower levels of *Acidaminococcaceae*, *Flavonifractor*, *Lachnoclostridium*, *Tyzzerellasubgroup-4*, and unidentified *Lachnospiraceae*, and higher levels of *Clostridium* compared to TD	*Clostridium ↑*	*Acidaminococcaceae↓* *Lachnospiraceae ↓* *Flavonifractor ↓* *Tyzzerellasubgroup-4 ↓* *unidentified Lachnospiraceae ↓*
Liu et al. (2019)	Altered gut microbiota and short chain fatty acids in Chinese children with autism spectrum disorder	Fecal	16 s rRNA Sample size: *N* = 50	30 ASD and 20 neurotypical	Levels of fecal acetic acid and butyrate and a higher level of fecal valeric acid in ASD subjects Decreased abundances of key butyrate-producing taxa (*Ruminococcaceae, Eubacterium, Lachnospiraceae and Erysipelotrichaceae*) Increased abundance of valeric acid associated bacteria (*Acidobacteria*) Enriched *Fusobacterium*, *Barnesiella, Coprobacter* and valeric acid-associated bacteria (*Actinomycetaceae*) and reduced butyrate-producing taxa in constipated autistic subjects	*Acidobacteria ↑* *Fusobacterium↑* *Barnesiella ↑* *Coprobacter ↑*	*Ruminococcaceae, ↓* *Eubacterium ↓* *Lachnospiraceae ↓* *Eubacterium ↓*
Ragusa et al. (2020)	Potential Associations Among Alteration of Salivary miRNAs, Saliva Microbiome Structure, and Cognitive Impairments in Autistic	Saliva	16 s rRNA + miRNA sequence Sample size: *N* = 80	53 ASD and 27 TD	In with ASD, miR-29a-3p and miR-141-3p were up-regulated, while miR-16-5p, let-7b-5p, and miR-451a were down-regulated The microbiome analysis of the same subjects showed that patients with ASD had an increased number of *Rothia*, *Filifactor*, *Actinobacillus*, *Weeksellaceae*, *Ralstonia*, *Pasteurellaceae*, and *Aggregatibacter*. In contrast, *Tannerella*, *Moryella*, and *TM7-3* exhibited a decrease in number	*Rothia ↑* *Filifactor ↑* *Actinobacillus ↑* *Weeksellaceae ↑* *Ralstonia ↑* *Pasteurellaceae ↑* *Aggregatibacter ↑*	*Tannerella ↓* *Moryella↓* *TM7-3↓*
Kang et al. (2020)	Distinct Fecal and Plasma Metabolites in with autism spectrum disorders and Their Modulation after Microbiota Transfer Therapy	Plasma; fecal	16 s rRNA Sample size: *N* = 38	18 ASD and 20 TD	Metabolite profiles in plasma and fecal samples were compared between subjects with ASD and a TD group before and after MTT treatment. The results indicate changes in the metabolite profiles after treatment Changes in metabolites represent a mechanism of gut microbiota-mediated gut-brain connectivity, providing credible clinical evidence for promising autism treatments and biomarkers	*/*	
Needham et al. (2021)	Plasma and Fecal Metabolite Profiles in Autism Spectrum Disorder	Plasma; fecal	Metabonomics Sample size: *N* = 231	130 ASD and 101 TD	Metabolites in plasma and feces differ between ASD and TD, and metabolite levels correlate with clinical behavioral scores Mitochondrial dysfunction may not only be a potential contributing factor to autism spectrum disorders but also a complication Steroid hormone levels are elevated in patients with autism spectrum disorders, and levels of lipid metabolites, and phenolic xeno-metabolites differ in ASD. Metabolites involved in oxidative stress are significantly associated with ADOS scores	*/*	
Cao et al. (2021)	Dysbiotic Gut Microbiota and Dysregulation of Cytokine Profile in Children and Teens with Autism Spectrum Disorder	Plasma	16 s rRNA Sample size: *N* = 86	45 ASD individuals and 41 healthy control subjects	Higher plasma levels of IL-2, IL-4, IL-5, IL-6, IL-10, TNF-α, TNF-β, and IFN-γ ASD gut microbiome is characterized by reduced levels of several beneficial microbiota, including *Bacteroides* and *Lachnospiraceae*	*/*	
Abdulhaq et al. (2021)	Tongue microbiome in children with autism spectrum disorder	Tongue	16 s rRNA Sample size: *N* = 63	25 children with ASD and 38 neurotypicals	Species richness and diversity did not significantly differ between the study groups Thirteen species and three genera were differentially abundant between the two groups Enrichment of *Actinomyces odontolyticus* and *Actinomyces lingnae* and depletion of *Campylobacter concisus* and *Streptococcus vestibularis* in the ASD group	*Actinomyces ↑* *odontolyticus ↑* *Actinomyces lignnae ↑*	*Campylobacter concisus ↓* *Streptococcus vestibularis ↓*
Wan et al. (2022)	Underdevelopment of the gut microbiota and bacteria species as non-invasive markers of prediction in autism spectrum disorder	Fecal	Metagenomic Sample size: *N* = 146	72 ASD and 74 TD	Age has the greatest effect on fecal flora in children with autism spectrum disorders, with no correlation to diet Children with ASD have been found to have an altered fecal microbial composition and increased bacterial abundance compared to TD children Children with ASD had an altered fecal microbial composition, increased bacterial abundance, and depletion of several neurotransmitter biosynthesis-related pathways in the intestinal microbiome compared to TD children Identified five bacterial species for disease prediction modeling	*/*	
Xu et al. (2022)	Leveraging Existing 16srRNA Microbial Data to Define a Composite Biomarker for Autism Spectrum Disorder	Fecal	16 s rRNA Sample size: *N* = 1,019	Data from 10 published research studies	No significant difference in fecal microbial diversity was found between the autism spectrum disorder group and the control group. However, differences in composition and structure were observed Diagnostic capabilities of microbial biomarkers determined by machine learning analysis, 12 genera were identified to distinguish autism spectrum disorders from controls	*/*	
Tong et al. (2022)	Implications of oral streptococcal bacteriophages in autism spectrum disorder	Fecal; plaque	Metagenomic Sample size: *N* = 52	26 ASD and 26 TD	Oral phageome’s abundance and α diversity are significantly reduced in ASD, streptococcal phages are abnormally abundant in it, and this is positively correlated with the severity of clinical manifestations of autism The association between the over-enrichment of *streptococcal* phage and ASD pathogenesis is specific	Streptococcal phage ↑	
Bundgaard-Nielsen et al. (2023)	Children and adolescents with attention deficit hyperactivity disorder and autism spectrum disorder share distinct microbiota compositions	Fecal; urine; blood	16 s rRNA Sample size: *N* = 96	32 ADHD; 12 ASD; 11 comorbid ADHD/ASD; 23 sibling controls; 17 non-related controls	The α and β diversity of gut microbiomes in ADHD and ASD are highly similar and different from those of unrelated controls Concentrations of the lipopolysaccharide-binding protein LBP are increased in a subset of patients with ADHD and ASD compared to TD children. These concentrations positively correlate with interleukin (IL)-8, 12, and 13 Gut Barrier and Immunomodulatory Disorders in Children with ADHD or ASD	*/*	
Li H. et al. (2023)	Multi-omics analyses demonstrate the modulating role of gut microbiota on the associations of unbalanced dietary intake with gastrointestinal symptoms in autism spectrum disorder	Fecal	16 s rRNA Sample size: *N* = 180	90 ASD and 90 TD	Children with ASD showed 11 instances of altered gut microbiome and 397 altered metabolites compared to TD children 10 metabolites produced by the gut microbiome that are associated with constipation and overall gastrointestinal symptoms. This highlights the role of the gut microbiome in the relationship between diet, microbiome, and gastrointestinal symptoms in ASD	*Turicibacter ↑* *Coprococcus 1 ↑* *Lachnospiraceae FCS020 group ↑* *Allobaculum ↑* *Eggerthellaceae ↑* *Coriobacteriales Incertae Sedis ↑* *Megasphaera* sp. *BS-4 ↑*	*Ruminiclostridium 6 ↓* *Actinomycetales ↓* *Tyzzerella ↓* *Clostridium* sp. *BR31 ↓*
Manghi et al. (2024)	Large-scale metagenomic analysis of oral microbiomes reveals markers for autism spectrum disorders	Saliva	Whole-genome sequenced Sample size: *N* = 7,812	2025 US families with children diagnosed with ASD	Oral microbiome composition can discriminate ASD subjects from neurotypical siblings ASD children with IQ < 70 also exhibit lower microbiome strain sharing with parents	*/*	
Evenepoel et al. (2024)	Oral microbiota in autistic children: Diagnosis-related differences and associations with clinical characteristics	Tongue	16 s rRNA Sample size: *N* = 120	80 autistic children and 40 typically developing peers	*Solobacterium*, *Stomatobaculum*, *Ruminococcaceae UCG.014*, *Tannerella* and *Campylobacter* were significantly more abundant in autistic Individual differences in microbiome composition might be involved in shaping the clinical phenotype of autism	*Solobacterium ↑* *Stomatobaculum ↑* *Ruminococcaceae UCG.014 ↑* *Tannerella ↑* *Campylobacter ↑*	
Tang et al. (2025)	Alterations of oral microbiota in young children with autism: Unraveling potential biomarkers for early detection	Plaque	16 s rRNA Sample size: *N* = 55	25 children with ASD and 30 age- and sex-matched typically developing	*Microbacterium flavescens, Leptotrichia* sp. *HMT-212, Prevotella jejuni, Capnocytophaga leadbetteri, Leptotrichia* sp. *HMT-392,* and *Porphyromonas* sp. *HMT-278* were identified in the oral microbiota of ASD children *Fusobacterium nucleatum sub*sp.*, Polymorphum, Schaalia* sp. *HMT-180, Leptotrichia* sp. *HMT-498, Actinomyces gerencseriae,* and *Campylobacter concisus* were identified in TD controls A diagnose model generated by random forest and leave-one-out cross-validation achieved an accuracy of 0.813	*Microbacterium flavescens ↑* *Leptotrichia* sp. *HMT-212 ↑* *Prevotella jejuni ↑* *Capnocytophaga leadbetteri ↑* *Leptotrichia* sp. *HMT-392 ↑* *Porphyromonas* sp. *HMT-278 ↑* *Fusobacterium nucleatum sub*sp. *↑*	*Polymorphum ↓* *Schaalia sp. HMT-180 ↓* *Leptotrichia sp. HMT-498 ↓* *Actinomyces gerencseriae ↓* *Campylobacter concisus ↓*
Li et al. (2026)	Multiomics analysis reveals the exacerbating effect of constipation on autism-related symptoms in children with autism spectrum disorder	Fecal	16 s rRNA + non-targeted metabolomics Sample size: *N* = 55	90 children with ASD (30 with constipation; 60 without)	Constipated ASD children exhibited more severe autism-related symptoms and alterations in four bacterial taxa-the phylum *Bacteroidetes*, the family *Barnesiellaceae*, and the genera *Alistipes* and *Bilophila*, plus 451 metabolites compared to non-constipated ASD children *Bacteroidetes*, *Alistipes*, and *Bilophila* exacerbated the relationship between constipation and autism-related symptoms Chenodeoxycholic acid, palmitic acid, glutaric acid, arachidonic acid, and choline were significantly associated with autism-related symptoms	*Bacteroidetes ↑* *Barnesiellaceae ↑* *Alistipes ↑* *Bilophila ↑*	

GS, Gastrointestinal Symptoms; FMT, Fecal Microbiota Transplantation; MTT, Microbiota Transfer Therapy; TNF, Tumor Necrosis Factor; IL, Interleukin; LPB, Lipopolysaccharide Binding Protein; IQ, Intelligence Quotient; ADHD, Attention Deficit Hyperactivity Disorder; DD, Nonautistic Developmental; ASD, Autism Spectrum Disorder; ADOS, Autism Diagnostic Observation Schedule; TD, Typically Developing.

In recent years, increasing attention has been given to the role of gut viral and fungal communities in ASD phenotypes, with studies providing preliminary evidence of their potential involvement in immune modulation, inflammation, and neurodevelopmental processes that intersect with ASD traits (Retuerto et al., 2024; Wan et al., 2024; Yuan et al., 2025; Shrivastav et al., 2026). However, oral viral and fungal components have received comparatively little attention, representing a significant gap in ASD oral microbiome research. The oral cavity hosts one of the most complex microbial ecosystems in the human body, comprising bacteria, fungi, archaea, and viruses (Baker et al., 2024), and oral bacteriophages dysbiosis may be linked to neurodevelopmental variation and immune modulation relevant to ASD (Tong et al., 2022; Manghi et al., 2024). Emerging work further highlights the concept of an oral-gut-brain axis, in which oral microbial metabolites, systemic inflammation, and neural processes interact, potentially contributing to neuroimmune dysregulation (De Angelis et al., 2013; Needham et al., 2021; Tong et al., 2022; Bundgaard-Nielsen et al., 2023). Despite these insights, oral fungal and viral communities remain comparatively under-characterized relative to bacterial constituents, representing a critical knowledge gap. Given the accessibility of the oral microbiome and its potential to reflect systemic immune states, profiling fungal and viral signatures offers an opportunity to identify noninvasive biomarkers for neurodevelopmental alteration. Metagenomic technologies enable comprehensive characterization of microbial taxa and functional pathways beyond the limitations of culture-based methods (Wensel et al., 2022), providing a platform to integrate taxonomic and metabolic features with neurodevelopmental phenotypes.

## 6 The potential of metagenomics in advancing microbial evidences for autism

Traditional microbiological research faces significant limitations, as approximately 99% of environmental bacteria cannot be cultured under laboratory conditions. A pivotal breakthrough occurred in 1985 when Lane et al. introduced 16S rRNA sequencing, enabling the identification of previously unculturable microorganisms (Lane et al., 1985). 16S amplicon sequencing involves PCR amplification of hypervariable regions of the bacterial 16S rRNA gene, facilitating identification and quantification of microbial species and assessment of community diversity. Despite its widespread use, this method primarily provides genus-level resolution with limited strain-level sensitivity, posing challenges for precise microbial identification. Additionally, variability across multiple hypervariable regions can yield inconsistent results, reducing data reliability (Poretsky et al., 2014; Durazzi et al., 2021). Furthermore, amplified regions are often hypervariable, making precise bacterial identification across species challenging. Owing to the high variability among bacterial species, relying on a single conserved region for differentiation is often difficult. Moreover, sequencing multiple 16S rRNA regions can produce inconsistent results, introducing biases that compromise data reliability (Chakravorty et al., 2007; Rintala et al., 2017; Sirichoat et al., 2021). Moreover, 16S rRNA sequencing offers limited functional insights, constraining the prediction of microbial community functions and impeding a comprehensive understanding of their interactions in diseases such as ASD (Mitra et al., 2011; Langille et al., 2013). These limitations necessitate advanced sequencing methods capable of providing deeper microbial characterization and functional analysis.

**Figure 3:**
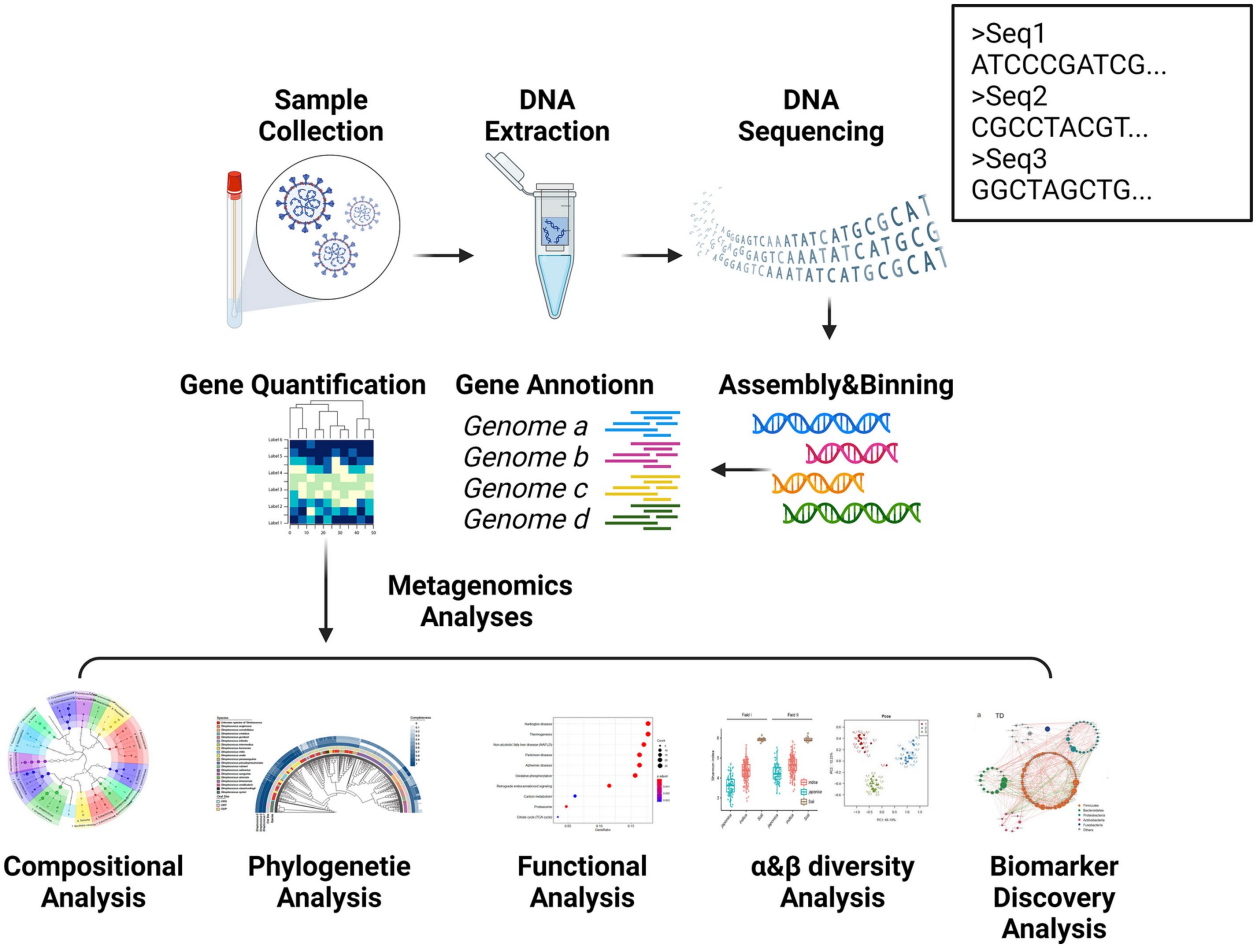
Process of metagenomic sequencing and subsequent analysis. Metagenomic sequencing technology allows for the capture of millions to billions of nucleic acid sequences in a single analysis, enabling the detection of a wide array of organisms, including novel pathogens present in clinical samples. This technology provides novel avenues for investigating the intricate interrelationship between the human microbiota and host well-being, as well as the functional contributions of these microbial communities to disease pathogenesis. Created with BioRender.com.

Metagenomic sequencing, introduced by Handelsman et al. (1998), represents a transformative advance by enabling direct genomic analysis of complex microbial communities without the need for culturing. This technology sequences all microbial DNA in a given sample, allowing comprehensive analysis of taxonomic composition and functional potential, including identification of virulence factors and metabolic pathways (Figure 3) (Quince et al., 2017). Unlike traditional culture-based methods and 16S sequencing, metagenomics provides detailed functional annotation, significantly enhancing the understanding of microbial community dynamics and host interactions (Mitra et al., 2011). Thus, metagenomics addresses key limitations of earlier sequencing methods and offers a powerful tool for elucidating microbial roles in complex diseases.

Clinical applications of metagenomics have greatly expanded the understanding of the role of the microbiome in systemic diseases. For example, in 2022, Palmer et al. utilized oral rinse samples and metagenomics to identify oral microbial biomarkers distinguishing pancreatic cancer patients from healthy individuals, indicating the potential of the oral microbiota as a disease biomarker (Petrick et al., 2022). Similarly, Nagat et al. analyzed the oral and intestinal microbiota in patients with pancreatic ductal adenocarcinoma (PDAC), establishing microbial profiles that are predictive of disease prognosis (Nagata et al., 2022). In 2023, Duan et al. employed metagenomics to investigate oral microbiome transmission in hypertension, demonstrating that saliva from hypertensive patients exacerbated angiotensin-II-induced hypertension in mouse models (Chen et al., 2023). These studies exemplify the capacity of metagenomics to elucidate microbiome–disease relationships, aiding in the development of diagnostic, preventive, and therapeutic strategies.

Metagenomics holds significant promise for investigating oral microbiome dysbiosis in individuals with ASD. By precisely characterizing microbial composition and functional capabilities, metagenomics can provide novel insights into how oral microbiota disturbances contribute mechanistically to ASD. Consequently, this approach presents opportunities for early diagnosis and targeted microbiome-based interventions, bridging microbiological findings with clinical applications in ASD management.

Overall, advancements in high-throughput sequencing, particularly metagenomics, provide critical insights into microbial composition and functional potential, significantly enhancing our understanding of microbial roles in ASD. We have to explore how these technological advances can be translated into practical diagnostic and therapeutic strategies, highlighting future research directions and clinical implications.

## 7 Exploring the potential of oral microbiome approaches for early autism diagnosis

In recent years, AI technology has become an increasingly important tool for disease prediction and diagnosis by enabling scalable analysis of large and complex datasets (Alhejaily, 2024). By applying machine learning algorithms, AI can identify clinically relevant patterns across heterogeneous data types, including structured clinical variables, medical images, and molecular profiles, that may be difficult to discern through manual review. These capabilities allow AI to provide early detection of diseases, predict patient outcomes, and assist in personalized treatment planning. Importantly, clinical decision-making is inherently multimodal, and multimodal machine learning offers a principled way to integrate complementary evidence streams, such as imaging, longitudinal signals, narrative reports, and structured clinical variables, within a single predictive framework (Krones et al., 2025). In radiology, integrating imaging with relevant clinical context has become a prominent direction, motivated by the need to improve diagnostic robustness while reducing interpretation workload and inter-reader variability (Simon et al., 2025). Beyond image-only models, multimodal diagnosis also includes joint modeling of medical images and text, such as aligning radiographs with accompanying reports for clinically meaningful retrieval and decision support, and developing image–text systems that generate draft reports while supporting downstream classification tasks (Jahanian et al., 2025; Liu et al., 2025). Similar integration is extending to precision medicine, where models that combine pathology images with molecular and clinical information have been proposed for prognostic and decision-support tasks, reflecting the complementary value of morphology, molecular profiles, and patient-level context (Hou et al., 2025). In parallel, multimodal learning is being explored to address a common bottleneck in omics translation, where cohort sizes are often limited; for example, transfer-learning frameworks that leverage large-scale electronic health records to support omics analyses have been reported to improve predictive modeling and patient stratification beyond omics-only approaches (Mataraso et al., 2025).

Among numerous biomarkers, the microbiome has received significant attention due to its extensive interactions with host health. With the improvement of AI analysis capabilities, it has become feasible to build predictive tools based on microbial communities. For example, machine learning has been applied to metagenomic data to identify reproducible microbial features associated with complex diseases such as inflammatory bowel disease, supporting disease classification and risk prediction (Lee et al., 2025). These developments motivate multimodal designs in settings where no single biomarker domain is sufficient, and where combining biological signals with clinical phenotyping can improve robustness and clinical interpretability (Krones et al., 2025). For example, models that integrate multi-omics data can capture functional patterns that are not apparent from a single assay, highlighting potential value for diagnosis- and prognosis-oriented modeling (Rozera et al., 2025). More recent work has combined microbiome features with conventional risk factors to build noninvasive screening models for atherosclerotic cardiovascular disease and other conditions, supporting the broader role of microbiome-informed decision support as an auxiliary approach (Sui et al., 2025).

However, identifying reliable and generalizable biomarkers for highly heterogeneous neurodevelopmental conditions such as ASD remains challenging. ASD-related behavioral signs often emerge in the first 2 or 3 years of life, yet formal diagnosis frequently occurs later in early childhood, underscoring a critical window for earlier identification and support. At present, ASD diagnosis relies predominantly on behavioral observation and standardized assessment instruments, and objective molecular criteria are not used in routine clinical practice. Espite advances in genetic research, genotype-based prediction has limited utility for routine clinical diagnosis given ASD heterogeneity (Cortese et al., 2025). Therefore, there is a clear need to explore accessible biomarkers that could support earlier and more objective risk stratification and referral decisions. Given the complexity of ASD, oral microbiome differences may offer complementary information when considered alongside behavioral data, although stronger conclusions will require more oral-sample studies and independent external validation. In recent years, the oral microbiome has been explored as a potentially noninvasive biomarker source in ASD research. Cross-sectional studies and systematic reviews have reported ASD-associated differences in oral microbial profiles, and several studies further suggest associations with quantitative clinical characteristics, supporting the possibility that oral signals may reflect ASD-related physiology (Evenepoel et al., 2024; Fakhruddin et al., 2025). Recent work has developed screening-oriented classifiers using oral microbiome features and reported encouraging internal-validation performance, supporting feasibility while highlighting the need for external validation. Collectively, these findings suggest that oral microbiome–informed models warrant further investigation as an adjunctive tool for ASD risk stratification, particularly if validated across independent cohorts (Tang et al., 2025). Integrating behavioral assessments with microbiome-derived features may better capture complementary aspects of ASD-related variability than either source alone (Solek et al., 2025). Metagenomic sequencing provides high-resolution microbial identification, which is critical for distinguishing subtle differences in microbial communities that may influence neurodevelopment. When combined with multimodal AI techniques that integrate behavioral patterns and microbiome data, such models may improve early risk stratification performance, although prospective validation is still needed (Temiz et al., 2025).

**Figure 4:**
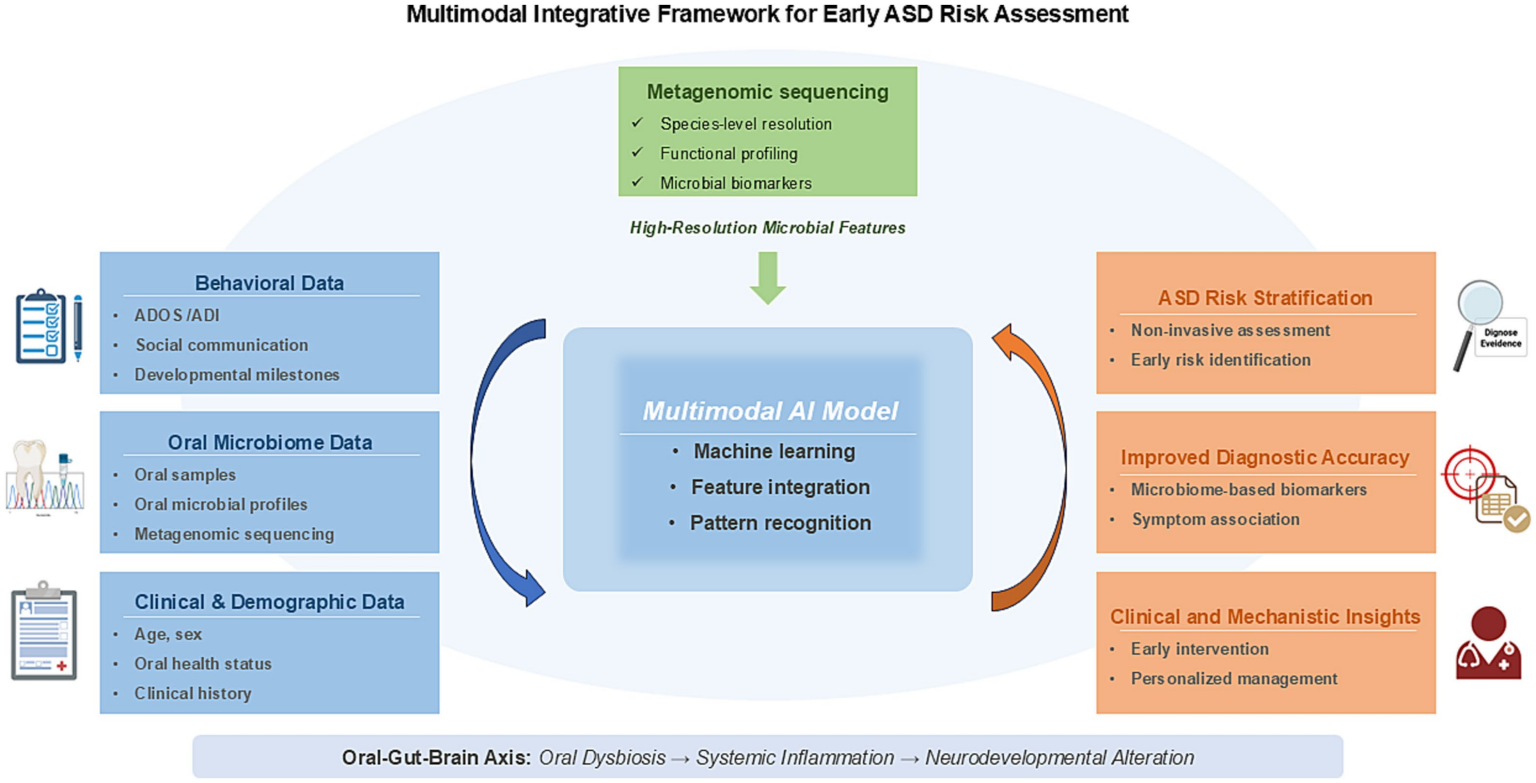
Multimodal AI model for early prediction and diagnosis of ASD. This schematic shows how behavioral measures, metagenomic oral microbiome features, and clinical variables can be combined within a multimodal AI model to support noninvasive early ASD risk prediction and diagnostic decision-making.

Building on evidence that oral microbial profiles differ between children with ASD and typically developing peers, we propose a preliminary multimodal, microbiome-informed AI framework for early ASD risk assessment and diagnostic support. The core objective is to integrate high-resolution oral metagenomic features with standardized behavioral assessment metrics within a unified computational model, so that microbial signals are interpreted in the context of clinically meaningful phenotypes. As illustrated in our schematic model, high-resolution oral microbial features derived from metagenomic sequencing can be integrated with behavioral assessments and relevant clinical/demographic information within an AI-enabled computational framework to enable feature integration and cross-domain pattern discovery (Figure 4). Large-scale salivary metagenomic analysis in family-based cohorts has demonstrated the feasibility of population-scale oral profiling and reported modest discriminative ability between ASD and neurotypical siblings, supporting feasibility rather than clinical readiness of high-resolution oral features as a scalable signal source (Manghi et al., 2024). In younger children, plaque-based profiling offers a complementary and relatively stable oral niche, and a preschool cohort study reported that plaque community composition carried discriminative potential, with a random forest classifier achieving 0.813 accuracy under leave-one-out cross-validation and strong ROC performance (Tang et al., 2025). Beyond single-sample-type evidence, associations between oral microbiome features and quantitative diagnostic characteristics have been reported and were not significantly driven by lifestyle differences, reinforcing the rationale for integrating microbiome readouts with behavioral phenotyping rather than treating microbial signals in isolation (Evenepoel et al., 2024). Additional oral sites may broaden practical options for child-friendly sampling and potentially improve robustness; for example, tongue-coating microbiota has been reported to differ between ASD and typically developing children and has been explored in predictive models when combined with clinical features (Zhong H. J. et al., 2025). Saliva-focused pilot work and earlier oral activity profiling studies also support the general plausibility of ASD-associated oral signatures, although heterogeneity across cohorts and platforms remains a major constraint for translation (Forsyth et al., 2020).

Our framework draws on complementary information sources that map directly onto the model inputs. Metagenomic sequencing generates high-resolution oral microbial features, including species- or strain-level taxonomic signals and functional pathway potentials, enabling more informative representations than genus-level summaries. Standardized behavioral assessments provide phenotype-anchored signals that reflect clinically meaningful ASD domains and help constrain. Basic clinical and demographic information is incorporated to reduce confounding and improve transportability across cohorts. This information typically covers age and sex, oral health status and relevant clinical history, and key exposures such as diet-related characteristics and medication use that can systematically shape oral microbiome profiles. The computational core performs multimodal learning to fuse heterogeneous inputs, and the fusion strategy is selected to match data availability and missingness patterns, consistent with broader multimodal machine learning practice in healthcare (Krones et al., 2025). The primary output is a calibrated risk probability for stratification together with interpretable feature contributions, so the tool supports transparent decision-making and referral prioritization rather than replacing gold-standard clinical diagnosis. This positioning is consistent with recent multimodal ASD screening frameworks that integrate accessible signals for scalable early risk categorization (Bae et al., 2025). Clinical translation will require rigorous validation, because microbiome-based classifiers often perform well in internal validation yet show reduced performance in cross-cohort evaluation. Standardized sampling and processing, explicit confounder control, and external validation in independent cohorts are therefore essential (Li P. et al., 2025). In principle, such an approach may support noninvasive ASD risk stratification in early childhood and help prioritize timely follow-up and intervention, while recognizing that prospective longitudinal studies are needed to establish real-world clinical utility.

## 8 Discussion

In this narrative review, we synthesize clinical and metagenomic evidence on ASD-associated oral microbiome alterations within an oral-gut-brain axis framework. Across studies, oral microbial differences between ASD and typically developing children are consistently reported, although the specific taxa and directions of change vary across cohorts and oral niches (Fakhruddin et al., 2025). A key explanation for this variability is oral site specialization: saliva, dental plaque, and tongue coating are distinct ecological habitats shaped by different local conditions and host factors (Mark Welch et al., 2020). In addition, ASD-related dietary selectivity, oral hygiene challenges, medication exposure, and caries or plaque status can shift oral community structure and may differ systematically between ASD and control groups (Como et al., 2021). To improve traceability and cross-study synthesis, future work should report sampling procedures, oral health assessment, and confounder handling more explicitly, in line with microbiome reporting recommendations (Mirzayi et al., 2021). Mechanistically, existing findings are compatible with links between oral dysbiosis, inflammatory signaling, and barrier perturbation, but most available evidence remains cross-sectional and associative, and causal direction cannot be inferred. From an application perspective, oral sampling is attractive for noninvasive screening support, but microbiome signals alone are unlikely to be sufficient given ASD heterogeneity. This motivates a multimodal framework that integrates high-resolution oral metagenomic features with standardized behavioral measures and basic clinical information to support early risk stratification and diagnostic decision support, with outputs framed as calibrated risk probabilities and interpretable feature contributions rather than replacements for gold-standard clinical diagnosis (Collins et al., 2015). A central challenge is transportability: microbiome-based classifiers often show optimistic performance in internal validation yet drop markedly under cross-cohort evaluation, highlighting the need for harmonized multi-center datasets, standardized processing, and rigorous external validation as part of a stepwise model-development pathway (Li M. et al., 2023). Finally, feasibility must be acknowledged in ASD populations. Saliva collection can be challenging for children with sensory sensitivities or limited cooperation, and studies should report acceptability and completion rates while considering alternative child-friendly oral sites such as plaque or tongue coating where appropriate (Janšáková et al., 2021; Zerman et al., 2022).

Overall, the evidence reviewed supports the presence of ASD-associated shifts in oral microbial ecology, while also underscoring that oral communities are strongly site-specific. Mechanistic links proposed along the oral–gut–brain axis remain largely inferential because most available datasets are cross-sectional and heterogeneous in sampling and analytical workflows. To move from association to clinically useful screening support, the field should prioritize harmonized study designs with transparent microbiome reporting, and prediction-model reporting that enables reproducibility and meaningful comparison across cohorts. Finally, the next step is to build harmonized multi-center oral datasets and use combined-cohort training with rigorous external validation, so that microbiome-informed multimodal models can become more transportable and clinically meaningful for early ASD risk stratification.
